# Genome-wide assessment of DNA methylation alterations induced by superovulation, sexual immaturity and in vitro follicle growth in mouse blastocysts

**DOI:** 10.1186/s13148-023-01421-z

**Published:** 2023-01-16

**Authors:** Laura Saucedo-Cuevas, Elena Ivanova, Anamaria-Cristina Herta, Felix Krueger, Katy Billooye, Johan Smitz, Gavin Kelsey, Ellen Anckaert

**Affiliations:** 1 Follicle Biology Laboratory (FOBI), UZ Brussel, Vrije Universiteit Brussel, Laarbeeklaan, Brussels, Belgium; 2 Epigenetics Programme, Babraham Institute, Cambridge, CB22 3AT UK; 3 Altos Labs, Bioinformatics Group, Cambridge, CB21 6GP UK; 4 Centre for Trophoblast Research, University of Cambridge, Cambridge, CB2 3EG UK; 5 Wellcome-MRC Institute of Metabolic Science-Metabolic Research Laboratories, Cambridge, CB2 0QQ UK

**Keywords:** Oocyte, Embryo, Superovulation, Imprinted genes, DNA methylation, In vitro follicle culture

## Abstract

**Background:**

In their attempt to fulfill the wish of having children, women who suffer from fertility issues often undergo assisted reproductive technologies such as ovarian stimulation, which has been associated with adverse health outcomes and imprinting disorders in children. However, given the crucial role of exogenous hormone stimulation in improving human infertility treatments, a more comprehensive analysis of the potential impacts on DNA methylation in embryos following ovarian stimulation is needed. Here, we provide genome-wide DNA methylation profiles of blastocysts generated after superovulation of prepubertal or adult mice, compared with blastocysts derived from non-stimulated adult mice. Additionally, we assessed the impact of the in vitro growth and maturation of oocytes on methylation in blastocysts.

**Results:**

Neither hormone stimulation nor sexual maturity had an impact on the low global methylation levels characteristic of the blastocyst stage or was associated with extensive DNA methylation alterations. However, we found hormone- and age-associated changes at specific positions but dispersed throughout the genome. In particular, we detected anomalous methylation at a limited number of CpG islands. Additionally, superovulation in adult mice was associated with alterations at the *Sgce* and *Zfp777* imprinted genes. On the other hand, in vitro culture of follicles from the early pre-antral stage was associated with globally reduced methylation and increased variability at imprinted loci in blastocysts.

**Conclusions:**

Our results indicate a minimal effect of ovarian stimulation of adult and prepubertal mice on the DNA methylation landscape attained at the blastocyst stage, but potentially greater impacts of in vitro growth and maturation of oocytes. These findings have potential significance for the improvement of assisted reproductive techniques, in particular for those related to treatments in prepubertal females, which could be crucial for improving human fertility preservation strategies.

**Supplementary Information:**

The online version contains supplementary material available at 10.1186/s13148-023-01421-z.

## Background

The use of assisted reproductive technology (ART) treatment is increasing worldwide with around 2.5 million reported cycles per year, and a total of 500,000 babies were born annually as of 2019 [1]. Within these technologies, in vitro fertilization (IVF) and, in particular, ovarian stimulation with exogenous hormones have been widely used for retrieving large numbers of mature oocytes, which increases the number of viable embryos produced per reproductive cycle and, therefore, the chances of conception [2]. As an alternative to reduce treatment burden, oocyte in vitro maturation (IVM) of cumulus–oocyte complexes retrieved from small- and medium-sized antral follicles in non- or minimally stimulated cycles eliminates the risk of ovarian hyperstimulation syndrome (OHSS) in polycystic ovary syndrome (PCOS) patients [3]. On the other hand, in vitro follicle culture (IFC) of early-stage follicles is an emerging ART with potential future applications for human oncofertility [4] when transplantation of cryopreserved ovarian cortical tissue is contraindicated because of a risk of reintroducing malignant cells.

Despite the fact that the majority of ART children are born healthy, there is concern about potential long-term consequences related to ovarian stimulation and in vitro culture techniques [5]. ART conception has been associated with adverse health outcomes such as preterm delivery, low birth weight, being small for gestational age or perinatal mortality [6]; cardiometabolic alterations [7]; and imprinting disorders, such as Beckwith–Wiedemann syndrome (BWS), Silver–Russell syndrome (SRS) and Prader–Willi syndrome (PWS) [8], although the extent to which ART procedures themselves or the underlying infertility of parents contribute is not fully understood. With the extended use of genome-wide approaches, more studies have focused on analyzing the global DNA methylation profile in children conceived by ART. For example, genomic epigenetic variation [9, 10] and differences at single genomic loci [11] associated with ART have been reported in cord blood by using whole-genome approaches to profile DNA methylation.

Ovarian stimulation, in vitro oocyte culture and in vitro culture of embryos occur at critical time points during the egg-to-embryo transition and coincide with global reprogramming of the epigenome and the establishment of epigenetic marks that persist into adulthood [12]. Therefore, the alterations found in ART children could be caused by a problem in the establishment of the maternal methylation profiles in the oocyte or their maintenance in the early embryo as a consequence of these techniques. For example, superovulation in mice has been reported to cause perturbations in the maintenance of methylation of the imprinted control regions (ICRs) of the *Snrpn*, *Kcnq1ot1* and *H19* loci in preimplantation embryos [13]. However, few studies have provided a comprehensive view of the effect of exogenous hormone stimulation on the oocyte/blastocyst epigenome.

Despite the significant progress made in ART over the last decades, a better understanding of the epigenetic processes underlying oocyte developmental competence is needed. In particular, those related to the dynamics of DNA methylation in prepubertal oocytes and resulting embryos could be crucial for improving human infertility treatment. Oocytes from (pre)pubertal girls are a potential source for in vitro growth and maturation in fertility preservation programs for cancer patients, but evidence suggests that oocytes from prepubertal females are inherently less competent [14]. For example, oocytes from prepubertal mice produce viable offspring after in vitro growth and maturation and fertilization [15]; however, the embryo developmental competence of these oocytes remains suboptimal compared to oocytes from adult females [16, 17]. Puberty is characterized by a drastic shift in neuroendocrine signaling and physical transformations [18], which are mirrored by changes in DNA methylation patterns in peripheral blood [19]. However, studies exploring sexual immaturity-associated DNA methylation profiles within the oocyte and the impact on the derived embryo are limited.

We previously evaluated DNA methylation in metaphase-II (MII) oocytes obtained from adult and prepubertal mice by ovarian superovulation, following in vitro follicle culture (IFC) from the early pre-antral stage and by natural ovulation. By applying whole-genome bisulfite sequencing (WGBS), we found that regardless of the treatment to mature the oocyte or the sexual maturity of the animals, the global DNA methylation pattern was largely conserved. In particular, no significant differences were found globally at genomic annotations such as gene bodies, intergenic regions, promoters, CpG islands (CGIs), or repetitive elements. Similarly, methylation at the hypermethylated and hypomethylated domains characteristic of the mouse oocyte genome was globally conserved. However, specific and recurrent localized differences in DNA methylation were found: IFC was associated with hypomethylation at a specific set of loci; methylation of superovulated prepubertal oocytes differed from that of superovulated adult oocytes, whereas, in contrast, oocytes from superovulated adult females differed very little from naturally ovulated oocytes [20]. Analysis of single blastocysts derived from these MII oocytes would now reveal whether the source, management or methylation landscape of oocytes has an impact on the epigenetic reprogramming in preimplantation embryos. In previous studies, we, and others, observed methylation abnormalities in imprinted genes in blastocysts derived from superovulated oocytes [21, 22]. However, such studies assessed a very limited number of imprinted loci and a more comprehensive analysis of the extent to which exogenous hormone stimulation affects the embryo epigenetic profile is needed. In the current study, we expand our previous findings by performing whole-genome DNA methylation analysis of mouse blastocysts with regard to the effects of superovulation and maternal age.

## Results

### Global DNA methylation profile is conserved in mouse blastocysts derived from superovulated adult and prepubertal oocytes

**Fig. 1:**
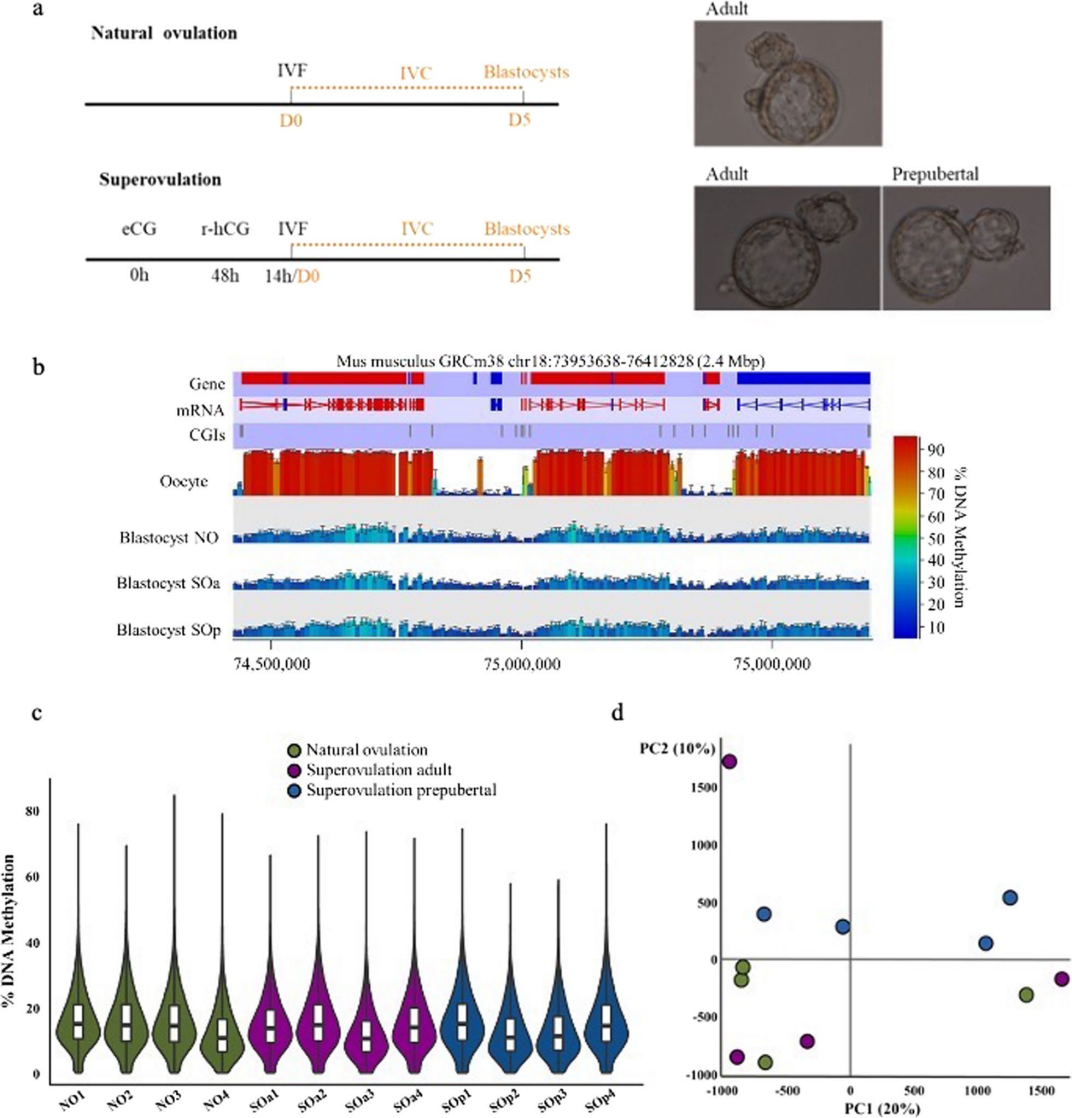
Global DNA methylation in mouse blastocysts from natural ovulation and superovulation groups. **a** In vivo matured MII oocytes were collected from adult mice (natural ovulation) and superovulated adult or prepubertal mice (superovulation). Superovulation was induced with an intraperitoneal injection of 2.5 IU (prepubertal) or 5 IU (adult) of eCG followed 48 h later by another intraperitoneal injection of the same dose of hCG. All MII oocytes underwent IVF. On Day 5 hatched blastocysts were collected. *Right panels*: Representative images of blastocysts on day 5 of embryo culture selected for PBAT. **b** Representative genome browser region showing the DNA methylation levels in naturally ovulated oocytes and NO, SOa and SOp blastocysts; the profile for each represents the merged PBAT data per group. Error bars indicate standard deviation. **c** Beanplots indicating whole-genome DNA methylation levels in individual blastocysts. The beanplots depict the density distribution of % CpG methylation of evaluated 100-CpG tiles common to all datasets (*n* = 206,059); within each beanplot, boxplot shows median value and 25–75th percentiles and whiskers show the lowest and highest observation. **d** Principal component analysis (PCA) of DNA methylation profiles for all 12 individual blastocysts. eCG, equine chorionic gonadotropin; hCG, human chorionic gonadotropin; IVF, in vitro fertilization; IVC, in vitro culture; NO, natural ovulation; SOa, superovulation adult; SOp, superovulation prepubertal. WGBS, whole-genome bisulfite-sequencing

In order to investigate the effect of superovulation and maternal age we generated mouse blastocysts (F1xF1; C57BL/6JxCBACa) derived from oocytes obtained after superovulation of adult (SOa; 8–10 weeks) and prepubertal (SOp; 23 days) mice and compared them with blastocysts produced with naturally ovulated oocytes (NO) from adult mice (Fig. 1a). Note that all zygotes were subject to the same in vitro culture conditions. Hatched blastocysts that looked developmentally most similar and presented the highest quality were collected on day 5 of in vitro culture (Fig. 1a). Post-bisulfite adaptor tagging (PBAT) DNA libraries were generated from six blastocyst per group to assess whole-genome DNA methylation profiles. However, two blastocysts from each of the categories NO, SOa and SOp and one from the IFCp group were excluded at the time of analysis based on a lower read coverage that could mean a major factor of the variation (Additional file 1: Figure S1). The sequencing output of all selected libraries is given in Additional file 2: Table S1. The yield of uniquely mapped and deduplicated reads for the libraries from these three groups varied between 2.33 × 10^7^ and 5.01 × 10^7^ in individual blastocysts, representing a minimal coverage of 52.6% of assessable genomic CpG sites. After merging the replicates by the experimental group, an average of between 2.90 × 10^7^ and 3.31 × 10^7^ uniquely mapped reads per group were obtained. In order to perform a detailed evaluation of the methylation across the genome, we defined non-overlapping tiles of 100 CpGs that segregated the genome into a total of 218,689. From these, we obtained 206,059 tiles with coverage in all 12 samples.

As a consequence of reprogramming, there is a genome-wide loss of DNA methylation that reaches the lowest level at the blastocyst stage. Consistent with this genome-wide erasure, we found extensive reduction in the methylated CpGs across the blastocysts’ genomes in each category compared to the bimodal pattern of methylation observed in oocytes (Fig. 1b). Global CpG methylation level (median methylation of 100-CpG tiles) was similar across all categories, with medians of 14.9% for the NO group (merged data), and 14.4% and 14.1% for SOa and SOp groups, respectively (Fig. 1c; Additional file 2: Table S1). Pearson correlation coefficients for every paired set across all 12 blastocysts indicated a similar range in correlations of 0.64–0.74 between replicates (Additional file 3: Figure S2). Accordingly, principal component analysis (PCA) did not show clustering of samples with respect to the experimental condition (Fig. 1d). We further assessed methylation levels at genomic features, such as intergenic regions and promoters, as well as mouse repetitive elements, finding comparable global CpG methylation levels across the three experimental groups (Additional file 4: Figure S3). There may appear to be a global effect on DNA methylation levels of LINE 1 and LTR-ERV1 repetitive elements; however, it is of limited magnitude since very few individual tiles reach the 10% cutoff difference. More specifically, SOp showed lower DNA methylation levels of LINE 1 and LTR-ERV1 repetitive elements than the SOa and NO conditions. However, after analyzing the individual elements that can be uniquely mapped and applying a 10% difference as a cutoff for biological significance, very few individual tiles were differentially methylated between conditions (Additional file 4: Figure S3).

### Superovulation results in limited DNA methylation alterations in blastocysts

**Fig. 2:**
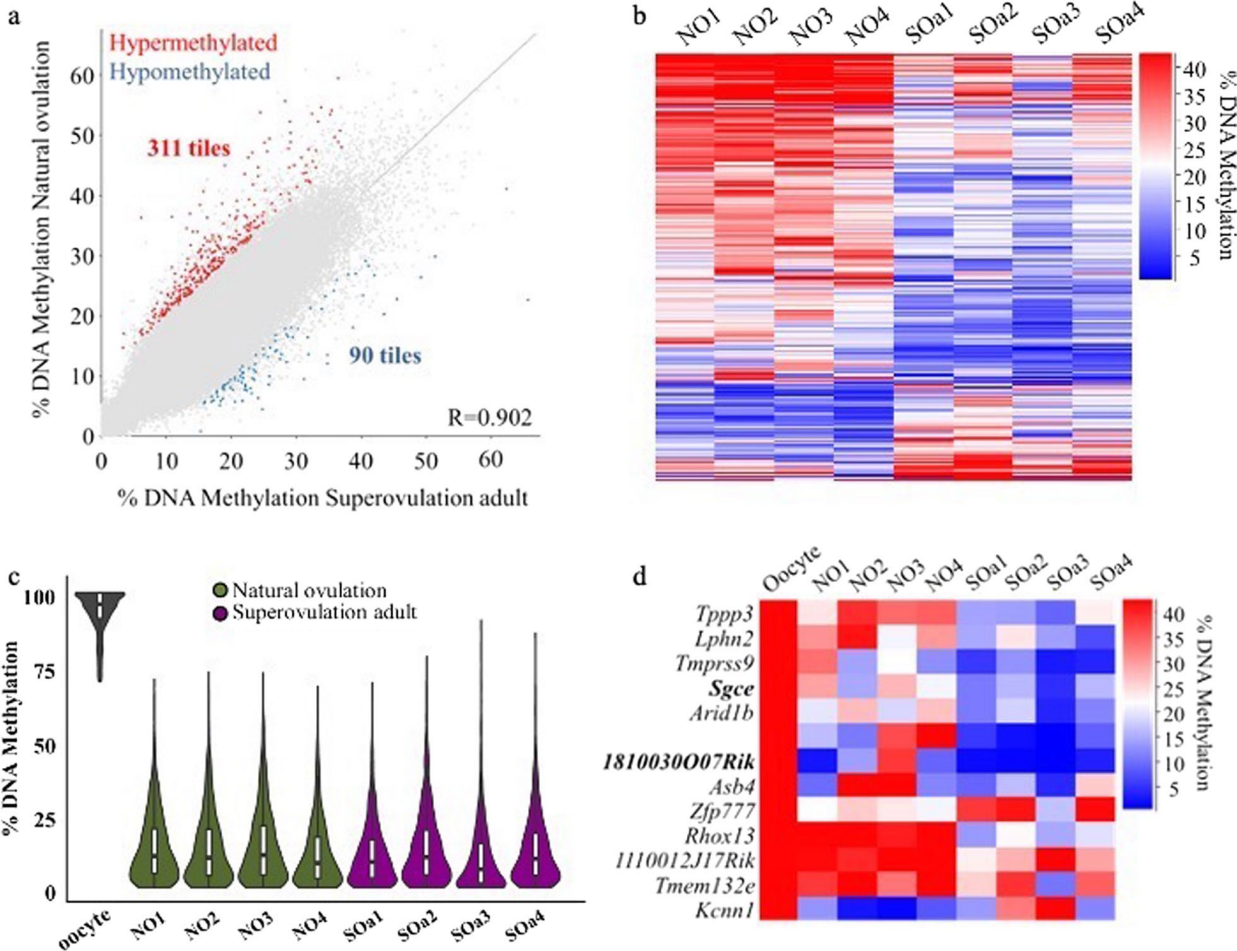
DNA methylation differences in mouse blastocysts from natural ovulation and superovulation adult conditions. **a** Scatterplot showing correlation between methylation levels of individual 100-CpG tiles common to all datasets (*n* = 206,059) in mouse blastocysts of the indicated two conditions, using data merged for the 4 samples per group. Differentially methylated tiles were determined by logistic regression analysis in SeqMonk (*p* < 0.05 corrected for multiple comparisons using Benjamini–Hochberg, methylation difference ≥ 10%). Red and blue indicate differentially methylated tiles that are hypermethylated or hypomethylated, respectively, in the NO group. **b** Heatmap showing the 100-CpG differentially methylated tiles identified between NO and SOa groups (*n* = 401). **c** Beanplots indicating methylation levels in blastocysts of the CGIs identified as methylated in MII oocytes (*n* = 1226); within each beanplot, boxplot shows median value and 25–75th percentiles and whiskers show the lowest and highest observation. **d** Heatmap showing the 13 differentially methylated CGIs identified between NO and SOa groups and the overlapping genes. CGI promoters are indicated in bold. NO, natural ovulation; SOa, superovulation adult; CGI, CpG island

We looked in more detail at the methylation profiles in blastocysts from the NO and aged-matched SOa groups in order to identify whether there were consistent alterations induced by hormonal stimulation of adult mice. We identified 401 100-CpGs tiles losing or gaining methylation (311 hypermethylated, 90 tiles hypomethylated in the NO group; Fig. 2a, b, Additional file 5: Table S5) after filtering for significantly different tiles (*p* < 0.05) with a difference ≥ 10%. In accordance with our previous results in oocytes [20], these genomic loci represented a very small percentage of the genome, only 0.2% of the total informative 100-CpG tiles assessed. These differentially methylated tiles were dispersed throughout the genome with no apparent enrichment over any specific gene or pathway.

We next evaluated CGIs specifically to test whether the subset of ~ 2000 CGIs described as highly methylated in the oocyte [23] would experience a similar loss of DNA methylation in both adult conditions, consistent with the expected genome-wide erasure over preimplantation stages. By taking as a reference the dataset generated from naturally grown and ovulated oocytes in our previous work [20], we filtered a set of 1226 oocyte-methylated CGIs common to all blastocysts in the NO and SOa groups and assessed the methylation ranges of these CGIs in the individual blastocysts. We observed a similar variation across blastocysts in both categories (Fig. 2c) and identified 13 differentially methylated CGIs (*p* < 0.05, methylation difference ≥ 10%; Fig. 2d, Additional file 5: Table S5). From these, only two were located at promoters: of the *Sgce* imprinted gene and the *1810030O07Rik* gene. The majority of the differentially methylated CGIs showed a loss of methylation in the SOa group; only two CGIs, overlapping the imprinted gene *Zfp777* and the gene *Kcnn1*, were more methylated in this group (Fig. 2d). These results would suggest a greater impact of hormone stimulation on the blastocyst methylation profile as only six CGIs were found altered (with a ≥ 20% difference) in SOa oocytes compared to NO oocytes in our previous work [20]. However, the few CGIs differentially methylated in oocytes, all of which were hypermethylated in SOa oocytes, were not among the altered CGIs detected in blastocysts. Altogether, this could suggest a different effect of superovulation at the oocyte and at the blastocyst stage. Furthermore, 58 CGIs were differentially methylated between the NO group and the SOp group (*p* < 0.05, methylation difference ≥ 10%), seven of which were also altered in the SOa group with the same pattern of methylation (Additional file 6: Figure S4). These results indicate a consistent effect, even if minor, of hormonal stimulation on DNA methylation regardless of sexual maturity of the animals.

### Maternal age causes minimal variations in blastocyst DNA methylation profiles

**Fig. 3:**
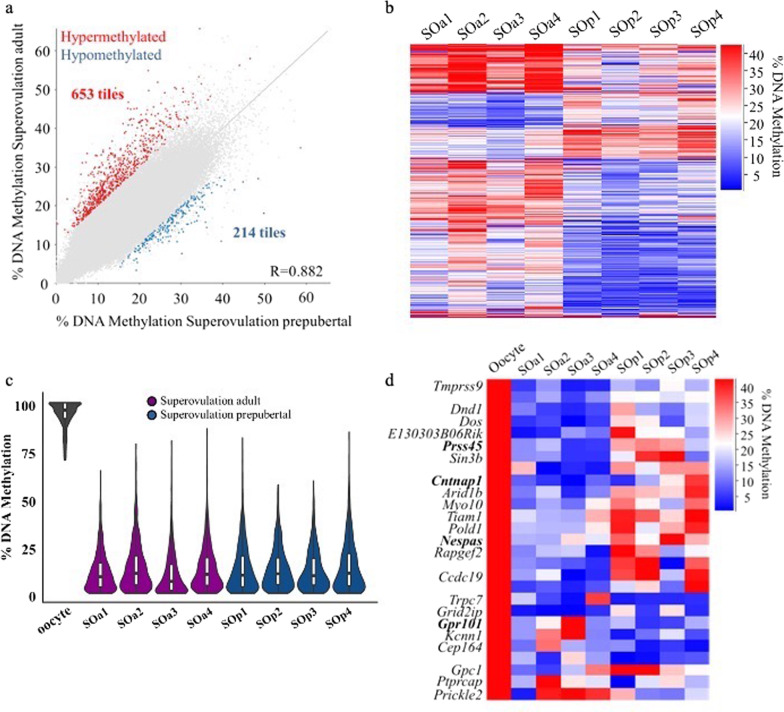
DNA methylation differences in mouse blastocysts from superovulation adult and superovulation prepubertal conditions. **a** Scatterplot showing correlation between methylation levels of individual 100-CpG tiles common to all datasets (*n* = 206,059) in mouse blastocysts of the indicated two conditions, using data merged for the 4 samples per group. Differentially methylated tiles were determined by logistic regression analysis in SeqMonk (*p* < 0.05 corrected for multiple comparisons using Benjamini–Hochberg, methylation difference ≥ 10%). Red and blue indicate differentially methylated tiles that are hypermethylated or hypomethylated, respectively, in the SOa group. **b** Heatmap showing the 100-CpG differentially methylated tiles identified between SOa and SOp groups (*n* = 867). **c** Beanplots indicating methylation levels in blastocysts of the CGIs identified as methylated in MII oocytes (*n* = 1226); within each beanplot, boxplot shows median value and 25–75th percentiles and whiskers show the lowest and highest observation. **d** Heatmap showing the 27 differentially methylated CGIs identified between SOa and SOp conditions and the overlapping genes. CGI promoters are indicated in bold. SOa, superovulation adult; SOp, superovulation prepubertal; CGI, CpG island

After identifying genomic regions likely affected by superovulation in blastocysts derived from adult females, we investigated whether hormone stimulation induced a different effect on the blastocyst methylation profile depending on female age, in light of the fact that we found that age was associated with far more methylation differences than superovulation in oocytes [20]. We identified 867 differentially methylated tiles (*p* < 0.05, methylation difference ≥ 10%;) after comparing the methylome of blastocysts from SOa and SOp mice (653 hypermethylated and 214 hypomethylated in the SOa group; Fig. 3a, b; Additional file 7: Table S6). Again, these differences accounted for a very small percentage of the genome (0.4%), in contrast to the more extensive differences in oocytes of the same categories (7.5% of the genome) [20]. It is important to note that the significant differences in methylation observed in the SOa:SOp comparison are not related to the NO group considered above, as SOa is the control group in this analysis. Similar to the previous comparison, differentially methylated tiles were randomly distributed across the genome with no association with specific genes or regions, and 531 of the 653 hypermethylated tiles were intergenic. However, differentially methylated tiles were similarly affected among the replicates, which would be a consequence of the statistical test (Fig. 3b). This would indicate a minimal age-related effect on DNA methylation in blastocysts following superovulation.

Specific analysis of the 1205 CGIs highly methylated in in vivo grown mouse oocyte [20] and common to all blastocysts in the SOa and SOp groups showed the expected general loss in methylation across the analyzed blastocysts (Fig. 3c). Statistical analysis identified 54 differentially methylated CGIs between the SOa and SOp groups (with a ≥ 10% difference, Fig. 3d, Additional file 7: Table S6); four were found overlapping the promoters of the genes *Prss45*, *Cntnap1* and *Grp101* and the imprinted gene *Nespas*. As observed in the NO:SOa comparison, there was a preferential decrease in methylation in the SOa group for these CGIs. Only two CGIs overlapping *Tmprss9* and *Kcnn1* genes where common to the lists NO:SOa and SOa:SOp. Since these two comparisons represent distinct treatments (donor age in the first case; superovulation in the second), so there is no reason to suppose that the same loci may be susceptible to the ‘treatments’. We also determined that the proportion of altered CGIs was greater at the blastocyst stage than in the oocyte: just eight CGIs were differentially methylated in oocytes [20] compared with 54 CGIs in blastocysts.

**Fig. 4:**
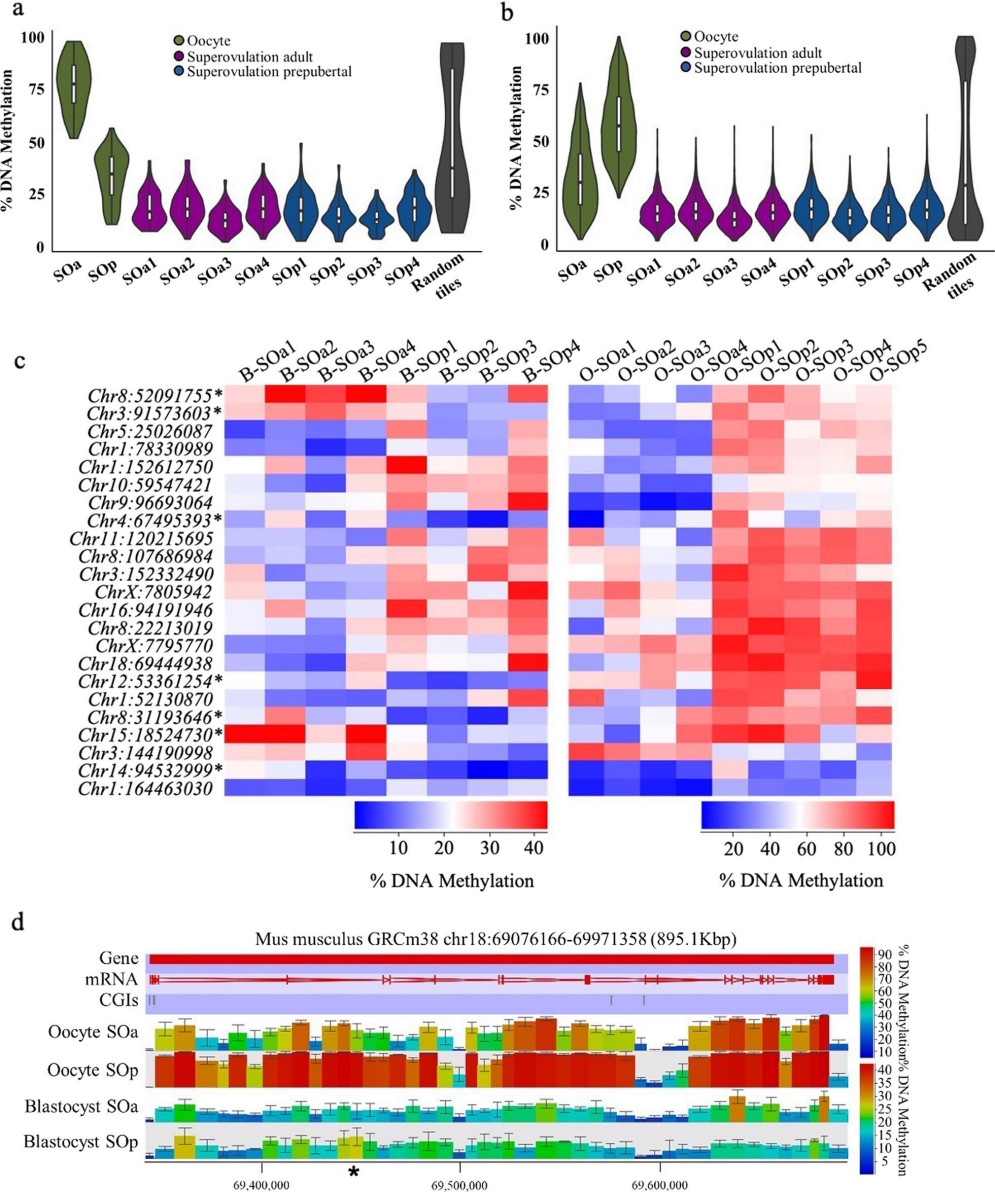
Limited persistence in blastocyst of methylation changes found in oocytes. **a**, **b** Beanplots indicating methylation levels in blastocysts of the regions identified as differentially methylated in MII oocytes that showed high methylation in the SOa (*n* = 48) (**a**) and SOp (*n* = 2031) (**b**) conditions. Within each beanplot, boxplot shows median value and 25–75th percentiles and whiskers show the lowest and highest observation. **c** Heatmaps showing the 23 differentially methylated 100 CpG-tiles in blastocyst (B) and in oocyte (O) identified between SOa and SOp conditions in oocytes that also scored differentially methylated in blastocysts. **d** Representative genome browser region showing the DNA methylation levels of the *Tcf4* gene. The asterisk indicates the remaining 100-CpG tile called differentially methylated in blastocysts. Each color-coded vertical bar represents the methylation value of the tile. The asterisk indicates a differentially methylated tile in the blastocyst. Error bars indicate standard deviation. SOa, superovulation adult; SOp, superovulation prepubertal; O, oocyte; B, blastocyst

Genome-wide analysis of SOa and SOp oocytes had detected methylation differences in 2079 100-CpG tiles [20]. To determine if these tiles remained differentially methylated to the blastocyst stage, we split them into 48 more highly methylated and 2031 tiles less methylated in SOa oocytes and determined their methylation range in blastocysts. Compared with random tiles, these tiles had a general reduction in methylation (Fig. 4a, b). Only 23 tiles were scored as having a methylation difference in blastocysts (Additional file 8: Table S7), of which 16 were differentially methylated in the same direction as in oocytes (Fig. 4c), but seven tiles displayed an opposite methylation change (Fig. 4c, asterisks). The majority of these tiles did not cluster over any specific gene. Only one overlapped a CGI (at the gene *Gripap1*) and another coincided with a promoter (at the gene *Vps36*). Overall, our study reveals that most of the differences in methylation between adult and prepubertal oocytes induced by hormonal stimulation are lost at the blastocyst stage. A similar analysis in the NO:SOa comparison identified only three tiles that remained differentially methylated to the blastocyst stage from the 565 identified in oocytes [20]. An example is shown in Fig. 4d. The most affected gene in the oocyte study was *Tcf4*, which contained 28 differentially methylated tiles [20], but only one was scored as differentially methylated in blastocysts (Fig. 4d, asterisk).

### Superovulation in adult mice is associated with altered DNA methylation at few imprinted loci

**Fig. 5:**
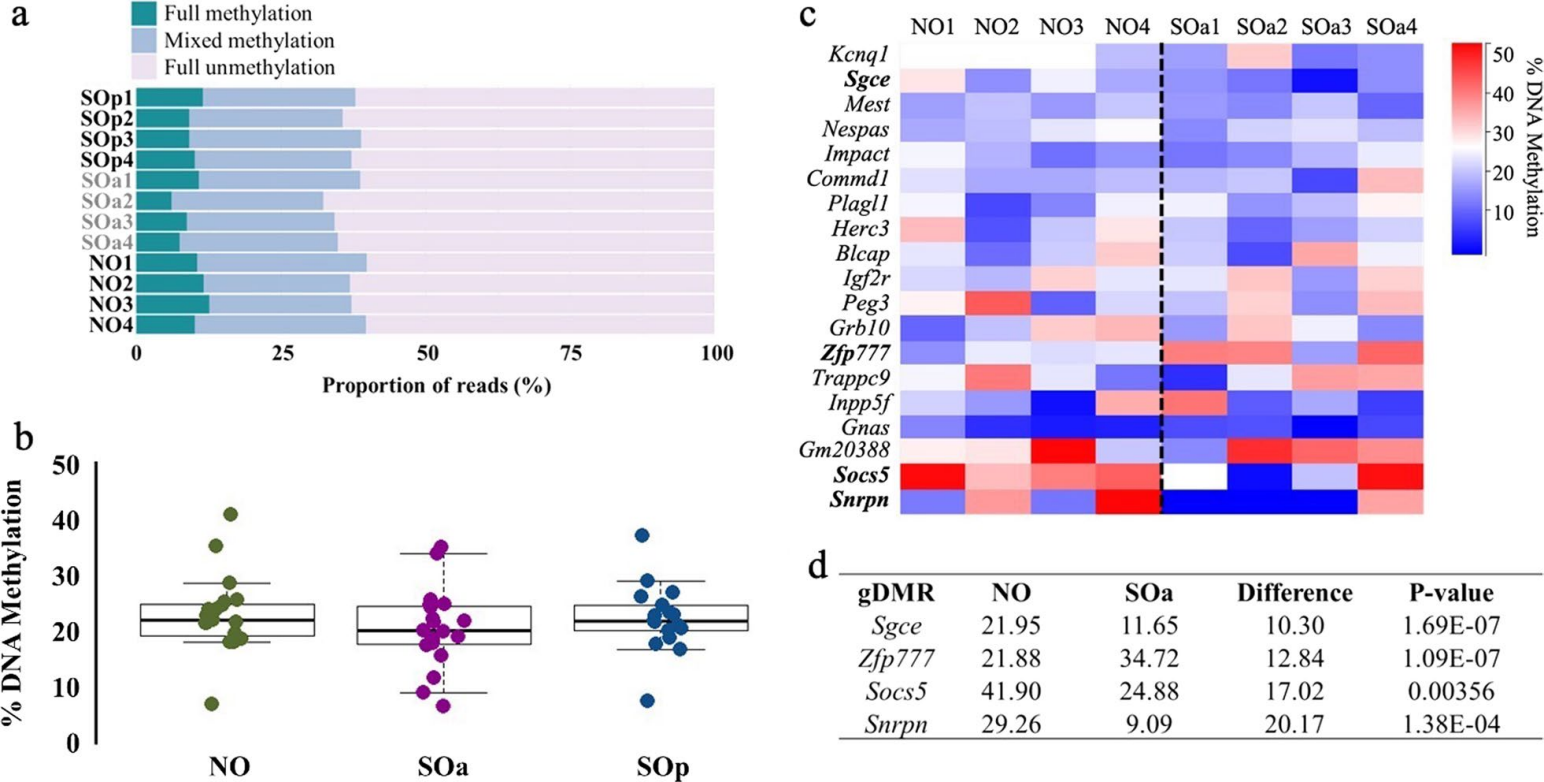
gDMR methylation in mouse blastocysts from natural ovulation and superovulation adult conditions. **a** Bar chart showing the proportion of reads that present full methylation, mixed methylation or full unmethylation for combined gDMRs in each individual blastocyst. **b** Strip chart and boxplot illustrating methylation at 17 gDMRs common to all blastocysts from the NO, SOa and SOp conditions. Each dot represents the combined methylation level of an individual gDMR in the blastocysts of the respective group. Boxplots show median value and 25–75th percentiles and whiskers show the lowest and highest observation. **c** Heatmap showing methylation levels at the 19 gDMRs common to all blastocysts within the NO and SOa conditions. Differentially methylated gDMRs are highlighted (determined by logistic regression analysis in SeqMonk; *p* < 0.05 corrected for multiple comparisons using Benjamini–Hochberg, methylation difference ≥ 10%). **d** Table indicating methylation and *p* values of the differentially methylated gDMRs. gDMR, maternally methylated germline differentially methylated region; NO, natural ovulation; SOa, superovulation adult; SOp, superovulation prepubertal

Germline differentially methylated regions (gDMRs) of imprinted genes are expected to maintain their methylation level after genome-wide reprogramming. In the oocyte, we demonstrated that imprinted DNA methylation acquisition is a robust process regardless of the treatment for oocyte maturation or the sexual maturity of the animals [20]. Here, we analyzed the methylation status of 19 maternally methylated gDMRs in the NO, SOa and SOp blastocysts. On average, individual blastocysts had a total number of ~ 3400 calls at CpG sites within these gDMRs. For the NO condition, gDMR methylation per blastocyst ranged between 20 and 23%, for the SOa group between 18 and 21% and for SOp, between 20 and 23% (Additional file 9: Table S2). We also determined methylation consistency at combined gDMRs in individual blastocysts, for which we evaluated the methylation level of reads that contained a minimum of 3 CpGs. > 60% of reads in each blastocyst were unmethylated and there were no significant differences in the proportions of fully methylated, partially or fully unmethylated reads between blastocysts in the three groups (Fig. 5a). Additionally, we assessed the methylation level of each gDMR after combining data within the NO, SOa and SOp groups. Overall, gDMRs across categories exhibited a similar range of methylation (Fig. 5b), in accordance with previously published work [23, 24].

We then evaluated methylation at individual gDMRs. Four had a statistically significant difference in the SOa group compared to NO: *Sgce*, *Zfp777, Soc5* and *Snrpn* (Fig. 5c, d). As a caveat, we note that *Soc5* and *Snrpn* were among the gDMRs with the lowest number of CpG calls (Additional file 10: Table S8), so sampling effects could contribute to greater variation in the data at these gDMRs. The *Sgce* gDMR displayed a significant loss of methylation in SOa blastocysts, and the *Zfp777* gDMR methylation gain (*p* < 0.05, ≥ 10% methylation difference).

### In vitro follicle culture results in altered blastocyst DNA methylation profiles

**Fig. 6:**
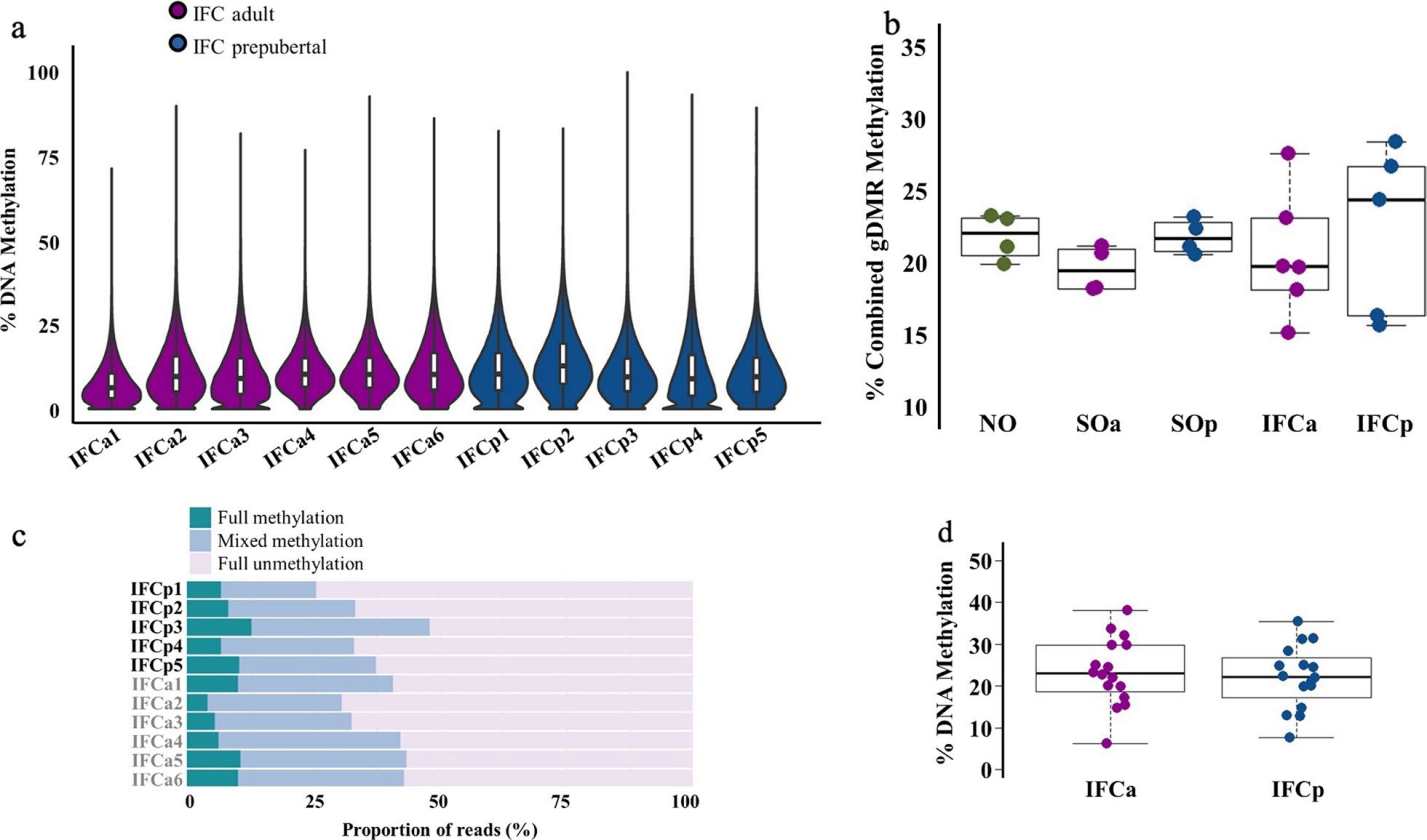
DNA methylation in mouse blastocysts derived from in vitro follicle culture oocytes. **a** Beanplots indicating whole-genome DNA methylation levels in individual blastocysts. The beanplots depict the density distribution of % CpG methylation of evaluated 100-CpG tiles common to all datasets (*n* = 193,363); within each beanplot, boxplot shows median value and 25–75th percentiles and whiskers show the lowest and highest observation. **b** Methylation at gDMRs in mouse blastocysts derived from NO, SOA, SOp, IFCa and IFCp conditions. Each dot represents the combined methylation level of the gDMRs of an individual blastocyst. Boxplots show median value and 25–75th percentiles and whiskers show the lowest and highest observation. **c** Bar chart showing the proportion of reads that present full methylation, mixed methylation or full unmethylation at combined imprinted gDMRs in each individual blastocyst. **d** Strip chart and boxplot illustrating methylation at 16 gDMRs common to all blastocysts from the IFCa and IFCp conditions. Each dot represents the combined methylation level of an individual gDMR in the blastocysts of the respective group. Boxplots show median value and 25–75th percentiles and whiskers show the lowest and highest observation. gDMR, maternally methylated germline differentially methylated region; SOa, superovulation adult; IFCa, in vitro follicle adult; IFCp, in vitro follicle culture prepubertal

IFC is an alternative method to mature oocytes; in the mouse, it is able to support the development of follicles from primordial or early pre-antral stages up to the preovulatory stage. IFC globally preserves the methylation landscape of oocytes and does not affect de novo methylation of imprinted genes [20], although we did find specific and consistent alterations in methylation in oocytes as a result of IFC [20]. In order to determine the consequences of IFC on methylation of blastocysts derived from in vitro cultured oocytes collected both from adult (IFCa) and prepubertal (IFCp) mice, we generated PBAT libraries individually from six IFCa and five IFCp blastocysts. The coverage of CpGs (≥ 1 read) in the individual blastocysts was lower (between 28.8 and 54.8%) than the NO, SOp and SOa datasets (Additional file 11: Table S3); this could be a consequence in part of the decreased number of cells observed in IFC blastocysts [25]. Pearson correlation coefficients of PBAT libraries for each paired set of analyzed blastocysts showed a weaker correlation between individual blastocysts with average pairwise correlation coefficients of 0.54 and 0.47 for IFCa and IFC, respectively (Additional file 12: Figure S5). This was lower than NO, SOp and SOa groups even if we downsized all datasets to be equivalent to the blastocyst with the lowest coverage (in the IFCp condition). This observation could suggest greater variation in methylation of the IFC-derived blastocysts. Moreover, the global methylation of individual IFC blastocysts was between 2 and 4% lower than the matched NO, SOp and SOa counterparts, the reduction being greater in the IFCa blastocysts (Fig. 6a). Overall, these results may suggest an adverse and non-specific effect of in vitro culture of pre-antral follicles on the DNA methylation profile of the resultant blastocysts.

We focused on the analysis of gDMRs in the IFC blastocysts. Consistent with the lower genome coverage of the IFCp and IFCa datasets, there was a lower average total of CpG calls at gDMRs (~ 1780), and a wider range in the combined gDMR methylation values per blastocyst (Fig. 6b, Additional file 13: Table S4). Five IFCa blastocysts and three IFCp blastocysts had < 50 CpG calls for more than three gDMRs (Additional file 10: Table S8). These effects could contribute to the observed higher variation. However, the IFC-derived blastocysts of both groups generally maintained fidelity of imprinted methylation (Fig. 6c) and exhibited similar variation in individual gDMR methylation levels to SOa blastocysts (Figs. 5b, 6d).

Looking at the gDMRs for which we obtained a more robust call quantification (> 50 total CpG calls), we found a significant gain of methylation at the *Mest* gDMR in IFCa compared to SOa blastocysts (*p* < 0.05, ≥ 10% methylation difference; Additional file 14: Figure S6). We also observed significant differences at the *Zfp777* gDMR; however, in this case, IFCa blastocysts displayed a methylation value similar to NO blastocysts (Fig. 5d), suggesting a gain of methylation due to hormone stimulation in adult mice. Regarding the possibility of an age-related effect in IFC, we found significant differences in methylation at the *Plagl1* and *Grb10* gDMRs (*p* < 0.05, ≥ 10% methylation difference; Additional file 14: Figure S6). However, methylation values in both IFCp and IFCa were very different from the averages in NO, SOa and SOp blastocysts for these gDMRs, which would suggest an adverse effect of IFC on the blastocyst DNA methylation profile. The *Herc3* gDMR also presented a significant difference in IFCa compared to SOa and IFCp (Additional file 14: Figure S6); but because of the low number of CpG calls for this gDMR in several IFC datasets, more analysis would be required to conclude an effect on its methylation. It is important to note that we found no difference in gDMRs methylation between the SOp and IFCp conditions. This would support our previous result that reported a similar level of DNA methylation at the *H19*, *Snrpn* and *Mest* imprinted genes in mouse blastocysts derived from IFCp [21].

## Discussion

We have performed unbiased, genome-wide DNA methylation profiling in blastocysts generated in vitro after hormone stimulation of prepubertal and adult mice in comparison to blastocysts derived from non-stimulated adults. We found no evidence that hormone stimulation caused substantial DNA methylation alterations either globally or preferentially at specific genomic annotations. However, we did identify a limited number of specific and reproducible hormone-associated epigenetic variation, which was dispersed throughout the genome. Similarly, we detected a minimal effect related to sexual maturity. Although the observed variation was not enriched over specific genes, both stimulation- and age-related abnormal methylation was observed at a small number of CGIs, and superovulation in adults was associated with alterations in two imprinted loci.

Although a number of studies indicate that ART may increase the incidence of adverse health outcomes in children by altering epigenetic mechanisms such as imprinting [8], it remains challenging to discriminate the influence of underlying infertility problems in the parents from the ART treatment itself [26]. In the past, many investigations have focused on the contribution of oocyte and embryo manipulation on imprinting alterations [13, 21, 22, 27–29], in view of the significance of correct DNA methylation of imprinted loci for offspring outcomes. However, given the scale and importance of epigenetic reprogramming during gametogenesis and embryonic development, it is vital to analyze possible DNA methylation alterations caused by ART at a genome-wide level. In this context, we recently reported the first whole-genome DNA methylation analysis of mouse MII oocytes obtained after IFC and following superovulation of prepubertal and adult mice [20]. Although our findings suggested limited impacts on DNA methylation of either the intervention used to generate mature oocytes or the age of the animals, analysis of blastocysts derived from these oocytes is essential to reveal the impact of gamete quality on epigenetic reprogramming as well as addressing the persistence of any effects induced in the oocyte.

Studies to date designed to determine the effects of superovulation on global DNA methylation in mouse zygotes and early embryos have not provided a consensus. For instance, loss or gain of nuclear 5-methylcytosine staining has been observed more often in two-cell mouse embryos derived from hormone-stimulated mice than in embryos obtained from naturally cycling females [30]. Other immunofluorescence studies have reported a reduction in global DNA methylation in zygotes following ovarian hyperstimulation [31, 32], and a faster genome-wide erasure of CpG methylation from zygote to 8-cell stage as a consequence of hormone treatment as assessed by WGBS [33]. Contrary to these above findings, Liang et al. described no significant effect on zygote global DNA methylation immunostaining after exposure to low or high doses of gonadotropins [34].

In our analysis, we did not detect extensive hormone-associated DNA methylation changes in blastocysts, suggesting that any gross epigenetic changes in zygote and early embryos reported in previous studies are lost by the blastocyst stage. In blastocysts derived from oocytes superovulated from adult females compared with natural ovulation, we identified only 0.2% of 100-CpG tiles as differing in methylation by ≥ 10%. We should note that our study comprised four blastocysts per group and that the PBAT libraries of individual embryos yielded coverage of genomic CpGs of 52–72%, so we are unable to detect more subtle changes with confidence, or those that might occur only in a proportion of embryos. In addition, a larger sample size would help to understand the variability in the level of methylation observed in CGIs and gDMRs among blastocysts of each condition. Nevertheless, that a subset of 7 differentially methylated CGIs of the 1226 oocyte-methylated analyzed was in common between blastocysts from adult and prepubertal superovulated groups would suggest reproducibility and loci that may be particularly sensitive to reprogramming or maintenance errors in the embryo.

The prepubertal ovarian environment experiences important transformations before and while entering puberty [35] mediated by sex hormones and intra‐ and extra‐ovarian factors [15, 17, 36, 37]. Therefore, it is worth considering that oocyte and follicle responses to signaling pathways and efficiency for DNA methylation acquisition may differ in prepubertal mice compared to young adults. This highlights the importance of studying prepubertal age models when considering the development of fertility preservation strategies for young girls. Accordingly, our study sought to determine the effects of sexual maturity on the methylation profile in blastocysts derived from superovulated oocytes. Significant differences in methylation between prepubertal and adult were greater than between superovulation and natural ovulation, accounting in this case for 0.4% of the genome. Nevertheless, this represents a reduced proportion of the analyzed tiles in this study compared to in oocytes. Additionally, few oocyte-derived methylation differences associated with the age of the animals at superovulation in the oocyte survived the post-fertilization demethylation process and were preserved to the blastocyst stage. Taken together, these results could indicate a minimal age-related effect in combination with exogenous hormone stimulation. However, these alterations could still affect the development of the placenta as it is known that maternal non-imprinted regions are involved in the regulation of placental development in mice and humans [38, 39]. To understand this fully, these loci will need to be interrogated in extraembryonic tissue in offspring generated from the various cohorts of blastocysts.

Contradictory findings have been reported regarding the effect of superovulation on imprinted DNA methylation in mouse oocytes and embryos. Some groups have reported no changes in methylation of *Igf2r*, *Peg1*, *Peg3*, *Plagl1*, *Snrpn*, *H19*, and *KvDmr1* in adult murine MII oocytes [29, 40, 41]. Accordingly, we found robust acquisition of DNA methylation at regulatory sequences of imprinted genes in adult mouse oocytes [27] and have subsequently extended and confirmed these results by genome-wide analysis of DNA methylation in prepubertal and adult MII oocytes [20]. Other studies have reported a loss of methylation on the maternal allele of *Snrpn* in morulae [42], of *Mest*, *Peg3*, *Snrpn* in blastocysts [22, 29], and gain of methylation on paternally methylated *H19* in blastocysts [22] from adult females. Loss of methylation at *Peg3* in postnatal brain tissue of juvenile offspring has been reported [43], but no alteration in the *Snrpn* and *H19* DMRs in mouse embryos from adult females has been described [44]. In previous work, we demonstrated that conventional ovarian stimulation in prepubertal mice produces loss of DNA methylation at regulatory sequences of three key imprinted genes at the blastocyst stage (*H19*, *Snrpn* and *Mest*) [21]. Our current findings provide a more comprehensive assessment of the extent to which superovulation might alter DNA methylation in imprinted regions in blastocysts. We found that the majority of 22 gDMRs we assessed were not affected as a consequence of superovulation or sexual maturity in mice; however, we did detect alterations at the *Sgce* and *Zfp777* gDMRs from superovulation of adult females. The zinc-finger protein 777 (ZFP777) plays a regulatory role in cell proliferation depending on cell density [45]; however, alterations in this gene have not been associated with the disease. Mutations in the maternally imprinted *SGCE* gene have been found to be the cause of developing myoclonus dystonia syndrome and psychiatric disorders [46], but no ART-associated defects in humans have been reported to date. Curiously, the *Sgce* and *Zfp777* gDMRs differed in opposite directions in response to superovulation in adult mice. It is important to note that not all imprinted genes are regulated through the same mechanisms. For example, deletion of maternal DPPA3 induces loss of imprinted DNA methylation at the *H19*, *Peg1*, *Peg3*, *Peg10* and *Rasgrf1*, but not of *Snrpn* and *Peg5* [47]. Similarly, ZFP57 and ZFP445 both contribute to the maintenance of gDMR methylation in early embryos but there is also some non-redundancy [48]. In the oocyte, we demonstrated that superovulation does not alter the methylation of CGIs, promoters, and gene bodies of key genes related to imprinting establishment and/or maintenance during preimplantation development such as *Dnmt1*, *Dnmt3a*, *Dnmt3L*, *Dppa3*, *Zfp57* and *Trim28* [20]. However, we did not study the effect of exogenous hormone stimulation on the oocyte transcriptome or proteome. Therefore, we cannot conclude whether variations in the expression of these genes could relate to the limited gDMR methylation differences we observed in early preimplantation embryos. In previous studies, a reduction in gene expression of the maternal-effect proteins BMP15, HDGF, DNMT1, DPPA3 and ZFP57 has been linked to superovulation [49]. Additionally, superovulation has been found to alter the amount of DNA methyltransferase proteins [50]. In fact, proteomic analysis of oocytes and blastocysts has demonstrated a distinct proteome profile associated with superovulation that affects phenotypic traits in mice [51]. Collectively, our findings, along with those from other studies, could suggest that superovulation has no impact on the establishment of DNA methylation at the gDMRs in oocytes; instead, it could disrupt maternal-effect gene products that are necessary during preimplantation for the maintenance of imprinting [40, 50].

Another aim of our study was to evaluate the potential longer-term effects of an established IFC system [21]. Previously, we showed that the diameter at the MII stage of IFC oocytes is smaller than in vivo grown mature oocytes [20], but genomic methylation acquisition in IFC oocytes was affected only to a limited extent. Here, we detected a 2–4% reduction in global methylation in IFC blastocysts, which was more pronounced with oocytes obtained from adult IFC; there was also an increased variability in the methylation of imprinted genes. Our results so far could highlight an adverse effect of IFC on methylation attained in blastocysts, in contrast to what we observed in IFC MII oocytes. However, this work is limited by the small number of blastocysts and cells per blastocyst analyzed, resulting in a low CpG coverage, making it difficult to quantify fully the effect of IFC on DNA methylation. As for other DNA methylation studies on blastocysts, some will not be of sufficient quality to sustain development to term and the proportion of the latter will be higher for the IFC condition [27], which may contribute to the increased variability in this group. Nonetheless, we provide a rigorous roadmap that opens the possibility for extensive future studies on the improvement in in vitro follicle culture conditions. In particular, the more pronounced loss of DNA methylation in the SOa group could reflect unsuited in vitro culture conditions as our IFC system has been optimized for pre-antral follicles derived from prepubertal animals and adult follicles may have different requirements. Given that oocyte maturation ensures all material required to undergo fertilization (reviewed in [52]), and that stored maternal factors in oocytes regulate oocyte differentiation into embryos during early embryonic development [53], it could be that the culture conditions alter the correct execution of transcriptional activity in the oocyte, which could have adverse effects on the embryo methylation landscape.

## Conclusions

In summary, we provide a genome-wide analysis of the DNA methylation profile of blastocysts derived from superovulated prepubertal and adult mice and blastocysts derived from non-stimulated adult mice. We show that both hormone stimulation and sexual maturity are associated with very limited alterations in the methylation of specific CGIs and imprinted genes, whereas IFC-derived blastocysts showed a decrease in global methylation and increased variability in imprinted DNA methylation. This study serves as a baseline to design markers for ART protocol optimization studies that can be targeted by DNA methylation assays such as pyrosequencing or droplet digital PCR (ddPCR). Future studies are needed to assess whether these specific methylation changes can be permanently propagated to the next generation or can have an impact on offspring health.

## Methods

### Generation and collection of mouse blastocyst samples

All animal experiments in this study were performed in accordance with a protocol approved by the Ethical Committee for the use of Laboratory Animal of the Vrije Universiteit Brussel. Mice were maintained and bred according to European and national standards for animal care. Mouse blastocysts, (F1xF1 C57BL/6xCBA), at the hatched stage were generated from [1] naturally ovulated (unstimulated) oocytes collected from adult mice (8–10 weeks) and [2] superovulated oocytes collected from adult (8–10 weeks) and prepubertal (23 days) female mice (Fig. 1).

#### Collection of unstimulated oocytes

Female estrous cycles were synchronized after three days of being in contact with male pheromones. Cumulus–oocyte complexes (COCs) were obtained by puncturing the largest antral follicles in the mouse ovaries with insulin syringe needles.

#### Collection of superovulated oocytes

Female mice were superovulated with an intraperitoneal injection of 2.5 IU (prepubertal) or 5 IU (adult) of equine chorionic gonadotropin (eCG; Folligon, Intervet) followed 48 h later by another intraperitoneal injection of the same dose of human chorionic gonadotropin (hCG; Chorulon; Intervet). Fourteen hours after hCG injection, oviducts are removed, and COCs were gently released from the ampulla.

#### Collection of oocytes from in vitro follicle culture (IFC)

Early pre-antral follicles of 110–130 µm in diameter were mechanically isolated from ovaries of 13-day-old and 8–10-week-old females and cultured in vitro for 10 days. The follicle culture medium consisted of α-minimal essential medium (Invitrogen) supplemented with 5% heat-inactivated fetal bovine serum, 5 µg/ml of insulin, 5 µg/ml of transferrin, 5 ng/ml of selenium (ITS; Sigma-Aldrich), and 10 IU/L of recombinant follicle-stimulating hormone (r-FSH; Gonal-F®, Serono) and 10 IU/L of recombinant luteinizing hormone (added once at the start of culture; r-LH, Luveris, Merck Serono). Follicles were individually cultured until the antral stage in an incubator at 37 °C, 100% humidity, and 5% carbon dioxide in air. At the end of Day 9, an ovulatory stimulus was given with 1.2 IU/ml of recombinant human chorionic gonadotropin (r-hCG; Ovitrelle, Serono) supplemented with 4 ng/ml of recombinant epidermal growth factor (r-EGF) (Roche Diagnostics). Approximately 18 h after r-hCG/r-EGF administration (Day 10) cumulus–oocyte complexes (COCs) containing MII oocytes were available for denudation with hyaluronidase.

#### In vitro fertilization (IVF)

All blastocysts were obtained after IVF and in vitro culture. Mature sperm was collected from the epididymal cauda of adult male F1 mice and incubated in M16 medium supplemented with 3% BSA and 1% nonessential amino acids (NEAA; 100 × ; Gibco) at 37 °C in 5% CO_2_ for 1 h to undergo capacitation. Once motile sperm were selected and counted, they were added to the COCs for 2 h and 30 min at 37 °C in 100% humidity, 5% CO_2_ and 6% O_2_. The fertilized oocytes were cleaned and transferred to embryo culture dishes in the same incubation conditions as mentioned above. The in vitro embryo culture medium contained M16 medium supplemented with NEAA (10 µl/ml) and essential amino acids (EAA; 50 × ; 20 µl/ml; Gibco). Cleavage was checked 24 h after IVF; and on Day 5 blastocyst development was evaluated and hatched blastocysts (~ 100 cells) that looked developmentally most similar and presented the highest quality were selected for further analysis.

### PBAT sequencing library preparation

Genome-wide DNA methylation profiles were generated according to the modification of the post-bisulfite adaptor tagging (PBAT) method as previously described [54]. Six blastocysts per condition were individually collected and lysed in 10 μl of RLT buffer (Qiagen). DNA was purified using solid phase reversible immobilization (SPRI) beads and converted with bisulfite reagent using EZ DNA Methylation-Gold kit (ZYMO Research, D5005). DNA was eluted in EB buffer and individual blastocyst PBAT libraries were prepared as outlined below. First-strand synthesis was performed using Klenow exo-tagged (New England Biolabs) and a biotinylated oligo I (5-[Btn]CTACACGACGCTCTTCCGATCTNNNNNNNNN-3), followed by Exo-I treatment (New England Biolabs) and SPRI purification. Purified DNA was incubated with Dynabeads Streptavidin M-280 beads (ThermoFisher Scientific) to capture biotinylated DNA and second-strand synthesis was carried out using reverse oligo II (5′-TGCTGAACCGCTCTTCCGATCTNNNNNNNNN 3’). Libraries were amplified using indexed iPCRTag reverse primers [55] with KAPA HiFi (Kapa Biosystems) DNA polymerase for 15 PCR cycles and purified using SPRI beads (Agencourt Ampure XP bead). Libraries were quantitated and quality control was checked using an Agilent High Sensitivity DNA kit and KAPA Illumina Library Quantification Kit for Illumina (Kapa Biosystems). Libraries were sequenced on the NextSeq500 platform in 100 bp single-end mode at the Babraham Institute Sequencing Facility.

### Methylation data analysis

Methylation data were trimmed using TrimGalore, deduplicated and mapped to the mouse genome assembly GRCm38 using Bismark (v.0.19.1) [56] and DNA methylation analysis was performed using the SeqMonk software package (v.1.42; Babraham Institute). Mapping was done onto the C57BL/6 J reference genome. However, mouse blastocysts F1xF1 C57BL/6xCBA were included in this work. C57BL/6 J and CBA/Ca genomes present sequence variants that could mislead the analysis. Specifically, Bismark infers C to T conversions that result from bisulfite treatment as being unmethylated and retained C sites as being methylated; therefore, C > T genetic variants could be erroneously called as unmethylated. To solve this issue, high-quality CBA/Ca SNPs from the Mouse Genomes Project (https://www.sanger.ac.uk/science/data/mouse-genomes-project) were used as a reference. Only 0.988% of genomic CpG sites coincided with a C > T SNP in the CBA/Ca genome. To obtain an unbiased analysis of the processed data, probes were defined as tiles of 100-CpGs where at least 10 CpGs are covered. Only informative tiles that were common to all replicates were included in the analysis. Two blastocysts from each of the categories NO, SOa and SOp and one from the IFCp group were excluded from the analysis based on low read coverage (Additional file 1: Figure S1). To define differentially methylated regions (DMRs), logistic regression was applied, with *p* < 0.05 after correction for multiple comparisons with the Benjamini–Hochberg analysis with an absolute methylation difference cutoff of 10%. Part of the genomic locations were defined using feature annotations available in SeqMonk. Promoters were considered − 1500 to + 500 bp around transcription start sites obtained from the mmEPDnew, the *Mus musculus* curated promoter database; CGI and gDMR features were called based on previously published genomic coordinates [23, 57]; for gene body + 500 bp to the end of the mRNA/gene was considered and intergenic regions were defined after excluding promoters; and gene bodies. Enrichment analysis was done using the Gene Ontology project website (http://geneontology.org/).

## Supplementary Information

**Additional file 1**. **Figure S1**: **a** Sequence outputs per individual blastocyst in the categories Natural ovulation, Superovulation adult, Superovulation prepubertal, In vitro follicle adult and In vitro follicle prepubertal. Blastocysts that were excluded from the analysis are highlighted in red. **b** Principal component analysis (PCA) of DNA methylation profiles for all 30 individual blastocysts. Outliers are highlighted in red. **c** Pairwise Pearson correlation matrix for individual blastocysts pairs after exclusion of LSC8 and LSC14 samples. 100 CpG window size tiles, n=193304 tiles; DNA methylation values between 0 and 100 in all 28 blastocysts; value of 1 is an ideal correlation.

**Additional file 2**. **Table S1:** Sequence outputs, global DNA methylation and CpG coverage (>1 read) per individual blastocyst in the categories: Natural ovulation, Superovulation adult and Superovulation prepubertal.

**Additional file 3**. **Figure S2**: Correlation matrix showing pairwise Pearson correlation values for individual sample pairs, where value of 1.0 is an ideal correlation. 100 CpG window size tiles, n=206059 tiles. NO, natural ovulation; SOa, superovulation adult; SOp, superovulation prepubertal.

**Additional file 4**. **Figure S3**. Global DNA methylation level of informative probes in NO, SOp and SOa datasets at different genomic features. NO, natural ovulation; SOa, superovulation adult; SOp, superovulation prepubertal.

**Additional file 5**. **Table S5**. Differentially hypermethylated tiles with more than 10% methylation difference in Natural ovulation compared to Superovulation adult. Each tile contains information about the genome location, overlapping gene, Ensembl ID, gene description and methylation percentage in each sample.

**Additional file 6**. **Figure S4**. Heatmap showing the seven differentially methylated CGIs identified between NO and SOp groups also found in the NO:SOa comparison. NO, natural ovulation; SOa, superovulation adult; SOp, superovulation prepubertal.

**Additional file 7**. **Table S6**. Differentially hypermethylated tiles with more than 10% methylation difference in Superovulation adult compared to Superovulated prepubertal.

**Additional file 8**. **Table S7**. Conserved differentially methylated tiles from oocyte to blastocyst in the comparison superovulated adult vs. superovulated prepubertal

**Additional file 9**. **Table S2**. Numbers of methylated and unmethylated CpG calls for the combined set of germline differentially methylated regions (gDMRs) in each NO, SOa and SOp blastocyst.

**Additional file 10**. **Table S8**. Number of CpG calls per gDMR and per sample.

**Additional file 11**. **Table S3**. Sequence outputs, global DNA methylation and CpG coverage (>1 read) per individual blastocyst in the categories IFCp and IFCa.

**Additional file 12**. **Figure S5**. Correlation matrix showing pairwise Pearson correlation values for individual sample pairs, where value of 1.0 is an ideal correlation. 100-CpG window size tiles, n=206059 tiles. NO, natural ovulation; SOa, superovulation adult; SOp, superovulation prepubertal. NO, natural ovulation; SOa, superovulation adult; SOp, superovulation prepubertal; IFCa, in vitro follicle culture adult; IFCp, in vitro follicle culture prepubertal.

**Additional file 13**. **Table S4**. Numbers of methylated and unmethylated CpG calls for the combined set of germline differentially methylated regions (gDMRs) in each IFCa and IFCp blastocyst.

**Additional file 14**. **Figure S6**. **a**, **b** Heatmaps showing methylation levels at the 18 gDMRs common to all blastocysts within the conditions SOa and IFCa (**a**) and at the 16 gDMRs common to all blastocysts within the IFCa and IFCp conditions (**b**). Differentially methylated gDMRs are highlighted (determined by logistic regression analysis in SeqMonk; p < 0.05 corrected for multiple comparisons using Benjamini–Hochberg, methylation difference ≥ 10%). **c**, **d** Tables indicating methylation and p values of the differentially methylated gDMRs.

## Data Availability

The data underlying this article are available in Gene Expression Omnibus database (GEO) under the accession number GSE205097.
