# Supplementary material for: Genome-wide assessment of DNA methylation alterations induced by superovulation, sexual immaturity and in vitro follicle growth in mouse blastocysts
Source: Clin Epigenetics. 2023 Jan 16;15:9. doi: 10.1186/s13148-023-01421-z (PMC9843966; doi:10.1186/s13148-023-01421-z)
Supplement: Supplementary file 1 — Additional file 1. Figure S1: a Sequence outputs per individual blastocyst in the categories Natural ovulation, Superovulation adult, Superovulation prepubertal, In vitro follicle adult and In vitro follicle prepubertal. Blastocysts that were excluded from the analysis are highlighted in red. b Principal component analysis (PCA) of DNA methylation profiles for all 30 individual blastocysts. Outliers are highlighted in red. c Pairwise Pearson correlation matrix for individual blastocysts pairs after exclusion of LSC8 and LSC14 samples. 100 CpG window size tiles, n=193304 tiles; DNA methylation values between 0 and 100 in all 28 blastocysts; value of 1 is an ideal correlation. [file 13148_2023_1421_MOESM1_ESM.docx]

**Additional Files**

a

b

c


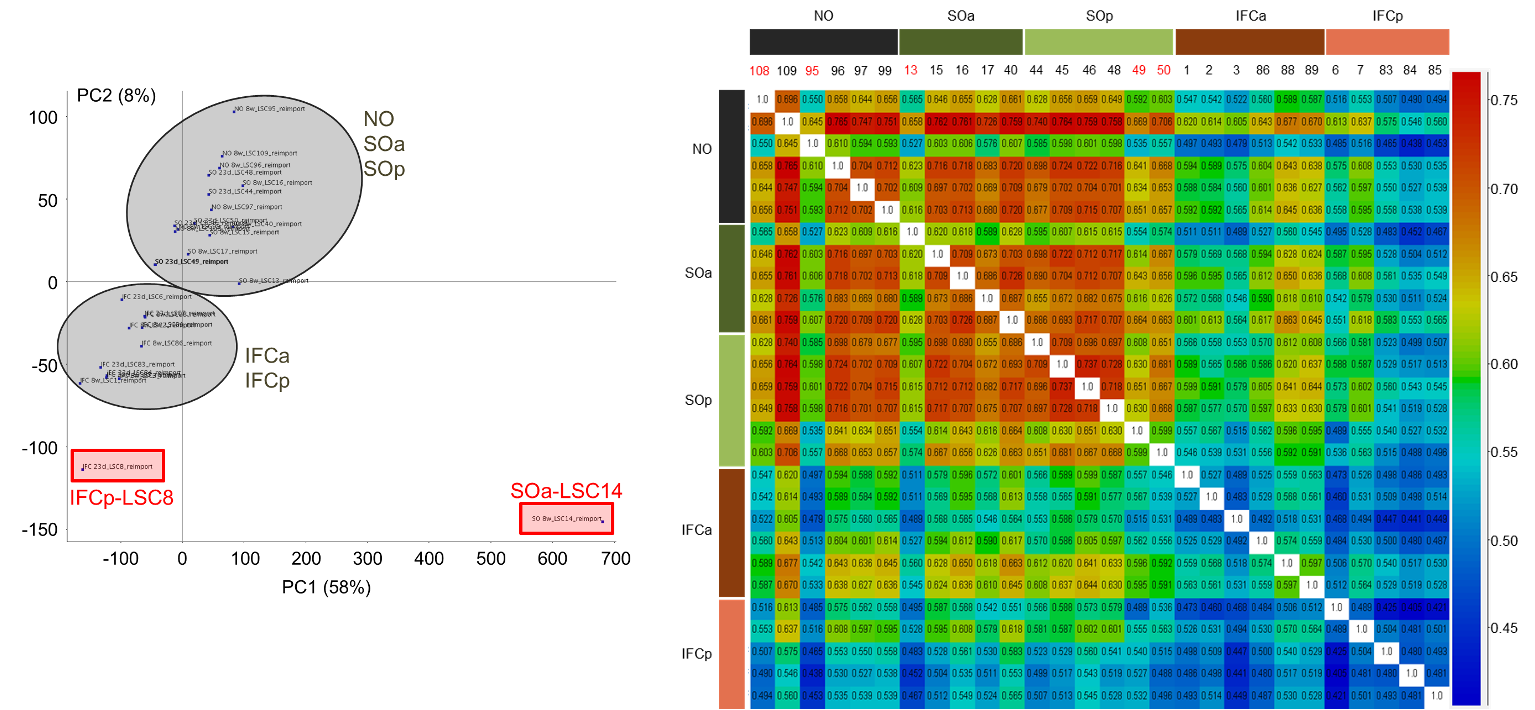


**Additional file 1: Figure S1.** (a) Sequence outputs per individual blastocyst in the categories Natural ovulation, Superovulation adult, Superovulation prepubertal, In vitro follicle adult and In vitro follicle prepubertal. Blastocysts that were excluded from the analysis are highlighted in red. (b) Principal component analysis (PCA) of DNA methylation profiles for all 30 individual blastocysts. Outliers are highlighted in red. (c) Pairwise Pearson correlation matrix for individual blastocysts pairs after exclusion of LSC8 and LSC14 samples. 100 CpG window size tiles, n=193304 tiles; DNA methylation values between 0 and 100 in all 28 blastocysts; value of 1 is an ideal correlation.

Libraries were excluded because of the substantially lower number of reads. Their inclusion in the analysis would necessitate down-sampling the data from the other libraries, which would mean losing a considerable number of data.
