# Supplementary material for: Genome-wide assessment of DNA methylation alterations induced by superovulation, sexual immaturity and in vitro follicle growth in mouse blastocysts
Source: Clin Epigenetics. 2023 Jan 16;15:9. doi: 10.1186/s13148-023-01421-z (PMC9843966; doi:10.1186/s13148-023-01421-z)
Supplement: Supplementary file 2 — Additional file 2. Table S1: Sequence outputs, global DNA methylation and CpG coverage (>1 read) per individual blastocyst in the categories: Natural ovulation, Superovulation adult and Superovulation prepubertal. [file 13148_2023_1421_MOESM2_ESM.docx]

**Additional file 2: Table S1.** Sequence outputs, global DNA methylation and CpG coverage (>1 read) per individual blastocyst in the categories: Natural ovulation, Superovulation adult and Superovulation prepubertal.
