# Supplementary material for: Genome-wide assessment of DNA methylation alterations induced by superovulation, sexual immaturity and in vitro follicle growth in mouse blastocysts
Source: Clin Epigenetics. 2023 Jan 16;15:9. doi: 10.1186/s13148-023-01421-z (PMC9843966; doi:10.1186/s13148-023-01421-z)
Supplement: Supplementary file 3 — Additional file 3. Figure S2: Correlation matrix showing pairwise Pearson correlation values for individual sample pairs, where value of 1.0 is an ideal correlation. 100 CpG window size tiles, n=206059 tiles. NO, natural ovulation; SOa, superovulation adult; SOp, superovulation prepubertal. [file 13148_2023_1421_MOESM3_ESM.docx]

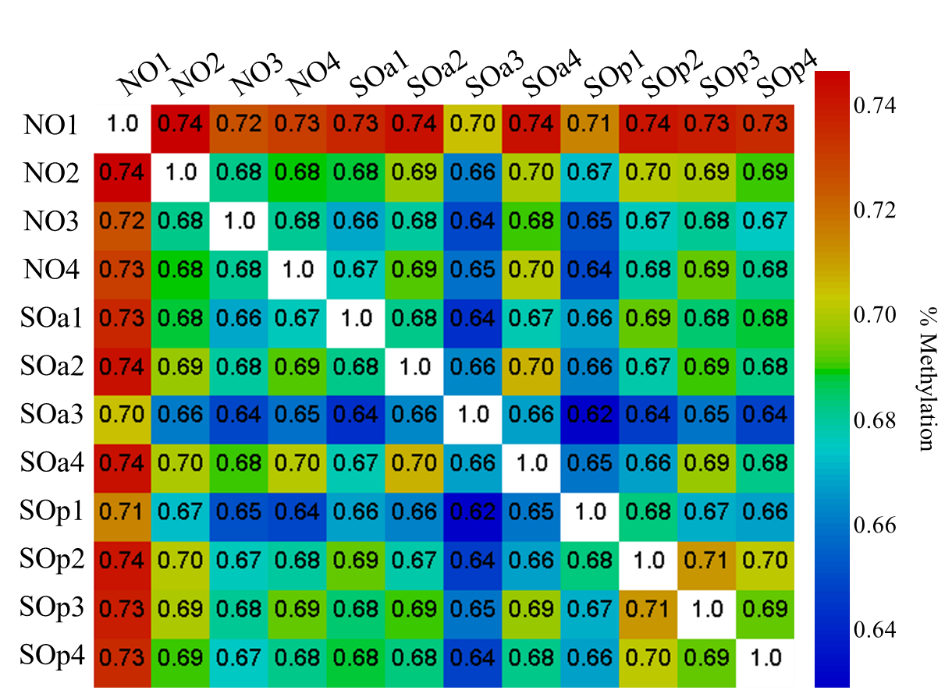


**Additional file 3: Figure S2.** Correlation matrix showing pairwise Pearson correlation values for individual sample pairs, where value of 1.0 is an ideal correlation. 100 CpG window size tiles, n=206059 tiles. NO, natural ovulation; SOa, superovulation adult; SOp, superovulation prepubertal.
