# Supplementary material for: Genome-wide assessment of DNA methylation alterations induced by superovulation, sexual immaturity and in vitro follicle growth in mouse blastocysts
Source: Clin Epigenetics. 2023 Jan 16;15:9. doi: 10.1186/s13148-023-01421-z (PMC9843966; doi:10.1186/s13148-023-01421-z)
Supplement: Supplementary file 4 — Additional file 4. Figure S3. Global DNA methylation level of informative probes in NO, SOp and SOa datasets at different genomic features. NO, natural ovulation; SOa, superovulation adult; SOp, superovulation prepubertal. [file 13148_2023_1421_MOESM4_ESM.docx]

**Additional file 4: Figure S3.** Global DNA methylation level of informative probes in NO, SOp and SOa datasets at different genomic features. NO, natural ovulation; SOa, superovulation adult; SOp, superovulation prepubertal.
