# Supplementary material for: Genome-wide assessment of DNA methylation alterations induced by superovulation, sexual immaturity and in vitro follicle growth in mouse blastocysts
Source: Clin Epigenetics. 2023 Jan 16;15:9. doi: 10.1186/s13148-023-01421-z (PMC9843966; doi:10.1186/s13148-023-01421-z)
Supplement: Supplementary file 6 — Additional file 6. Figure S4. Heatmap showing the seven differentially methylated CGIs identified between NO and SOp groups also found in the NO:SOa comparison. NO, natural ovulation; SOa, superovulation adult; SOp, superovulation prepubertal. [file 13148_2023_1421_MOESM6_ESM.docx]

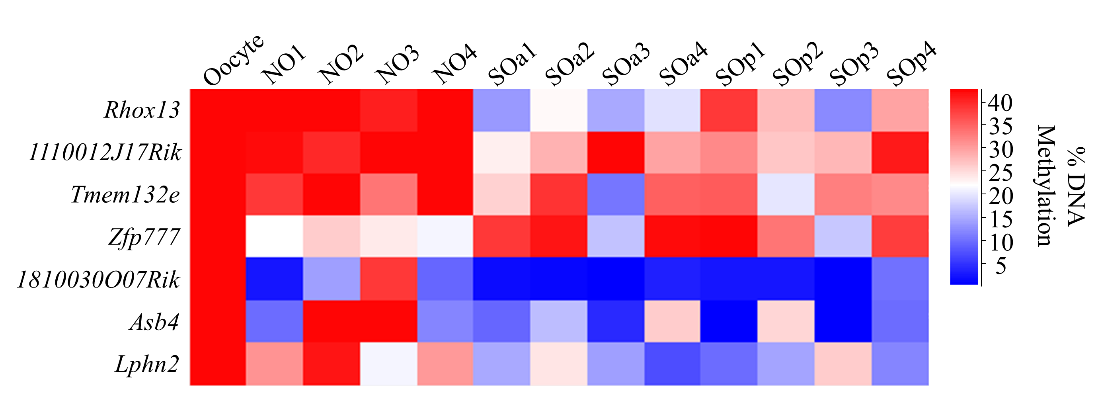


**Additional file 5: Figure S4.** Heatmap showing the seven differentially methylated CGIs identified between NO and SOp groups also found in the NO:SOa comparison. NO, natural ovulation; SOa, superovulation adult; SOp, superovulation prepubertal.
