# Supplementary material for: Genome-wide assessment of DNA methylation alterations induced by superovulation, sexual immaturity and in vitro follicle growth in mouse blastocysts
Source: Clin Epigenetics. 2023 Jan 16;15:9. doi: 10.1186/s13148-023-01421-z (PMC9843966; doi:10.1186/s13148-023-01421-z)
Supplement: Supplementary file 12 — Additional file 12. Figure S5. Correlation matrix showing pairwise Pearson correlation values for individual sample pairs, where value of 1.0 is an ideal correlation. 100-CpG window size tiles, n=206059 tiles. NO, natural ovulation; SOa, superovulation adult; SOp, superovulation prepubertal. NO, natural ovulation; SOa, superovulation adult; SOp, superovulation prepubertal; IFCa, in vitro follicle culture adult; IFCp, in vitro follicle culture prepubertal. [file 13148_2023_1421_MOESM12_ESM.docx]

**Additional file 8: Figure S5.** Correlation matrix showing pairwise Pearson correlation values for individual sample pairs, where value of 1.0 is an ideal correlation. 100-CpG window size tiles, n=206059 tiles. NO, natural ovulation; SOa, superovulation adult; SOp, superovulation prepubertal. NO, natural ovulation; SOa, superovulation adult; SOp, superovulation prepubertal; IFCa, *in vitro* follicle culture adult; IFCp, *in vitro* follicle culture prepubertal.
