# Supplementary material for: Genome-wide assessment of DNA methylation alterations induced by superovulation, sexual immaturity and in vitro follicle growth in mouse blastocysts
Source: Clin Epigenetics. 2023 Jan 16;15:9. doi: 10.1186/s13148-023-01421-z (PMC9843966; doi:10.1186/s13148-023-01421-z)
Supplement: Supplementary file 13 — Additional file 13. Table S4. Numbers of methylated and unmethylated CpG calls for the combined set of germline differentially methylated regions (gDMRs) in each IFCa and IFCp blastocyst. [file 13148_2023_1421_MOESM13_ESM.docx]

**Additional file 9: Table S4.** Numbers of methylated and unmethylated CpG calls for the combined set of germline differentially methylated regions (gDMRs) in each IFCa and IFCp blastocyst.
