# Supplementary material for: Genome-wide assessment of DNA methylation alterations induced by superovulation, sexual immaturity and in vitro follicle growth in mouse blastocysts
Source: Clin Epigenetics. 2023 Jan 16;15:9. doi: 10.1186/s13148-023-01421-z (PMC9843966; doi:10.1186/s13148-023-01421-z)
Supplement: Supplementary file 14 — Additional file 14. Figure S6. a, b Heatmaps showing methylation levels at the 18 gDMRs common to all blastocysts within the conditions SOa and IFCa (a) and at the 16 gDMRs common to all blastocysts within the IFCa and IFCp conditions (b). Differentially methylated gDMRs are highlighted (determined by logistic regression analysis in SeqMonk; p < 0.05 corrected for multiple comparisons using Benjamini–Hochberg, methylation difference ≥ 10%). c, d Tables indicating methylation and p values of the differentially methylated gDMRs. [file 13148_2023_1421_MOESM14_ESM.docx]

**Additional file 10: Figure S6**. (a, b) Heatmaps showing methylation levels at the 18 gDMRs common to all blastocysts within the conditions SOa and IFCa (a) and at the 16 gDMRs common to all blastocysts within the IFCa and IFCp conditions (b). Differentially methylated gDMRs are highlighted (determined by logistic regression analysis in SeqMonk; p < 0.05 corrected for multiple comparisons using Benjamini–Hochberg, methylation difference ≥ 10%). (c, d) Tables indicating methylation and p-values of the differentially methylated gDMRs.
